# Determination of ciprofloxacin in human plasma using high-performance liquid chromatography coupled with fluorescence detection: Application to a population pharmacokinetics study in children with severe malnutrition

**DOI:** 10.1016/j.jchromb.2010.11.032

**Published:** 2011-01-15

**Authors:** Simon N. Muchohi, Nahashon Thuo, Japhet Karisa, Alex Muturi, Gilbert O. Kokwaro, Kathryn Maitland

**Affiliations:** a Kenya Medical Research Institute (KEMRI)/Wellcome Trust Research Programme, Centre for Geographic Medicine Research (Coast), P.O. Box 230-80108, Kilifi, Kenya; b Department of Pharmaceutics and Pharmacy Practice, School of Pharmacy, University of Nairobi, P.O. Box 19676-00202 (KNH), Nairobi, Kenya; c African Centre for Clinical Trials, P.O. Box 2288-00202 (KNH), Nairobi, Kenya; d Consortium for National Health Research, P.O. Box 29832-00202 (KNH), Nairobi, Kenya; e Department of Paediatrics, Faculty of Medicine and The Wellcome Trust Centre for Clinical Tropical Medicine, Imperial College, Norfolk Place, London, W2 1PG, UK

**Keywords:** Ciprofloxacin, HPLC fluorescence detection, Plasma, Protein precipitation, Validation

## Abstract

Clinical pharmacokinetic studies of ciprofloxacin require accurate and precise measurement of plasma drug concentrations. We describe a rapid, selective and sensitive HPLC method coupled with fluorescence detection for determination of ciprofloxacin in human plasma. Internal standard (IS; sarafloxacin) was added to plasma aliquots (200 μL) prior to protein precipitation with acetonitrile. Ciprofloxacin and IS were eluted on a *Synergi Max-RP* analytical column (150 mm × 4.6 mm i.d., 5 μm particle size) maintained at 40 °C. The mobile phase comprised a mixture of aqueous orthophosphoric acid (0.025 M)/methanol/acetonitrile (75/13/12%, v/v/v); the pH was adjusted to 3.0 with triethylamine. A fluorescence detector (excitation/emission wavelength of 278/450 nm) was used. Retention times for ciprofloxacin and IS were approximately 3.6 and 7.0 min, respectively. Calibration curves of ciprofloxacin were linear over the concentration range of 0.02–4 μg/mL, with correlation coefficients (*r*^2^) ≥ 0.998. Intra- and inter-assay relative standard deviations (SD) were <8.0% and accuracy values ranged from 93% to 105% for quality control samples (0.2, 1.8 and 3.6 μg/mL). The mean (SD) extraction recoveries for ciprofloxacin from spiked plasma at 0.08, 1.8 and 3.6 μg/mL were 72.8 ± 12.5% (*n* = 5), 83.5 ± 5.2% and 77.7 ± 2.0%, respectively (*n* = 8 in both cases). The recovery for IS was 94.5 ± 7.9% (*n* = 15). The limits of detection and quantification were 10 ng/mL and 20 ng/mL, respectively. Ciprofloxacin was stable in plasma for at least one month when stored at −15 °C to −25 °C and −70 °C to −90 °C. This method was successfully applied to measure plasma ciprofloxacin concentrations in a population pharmacokinetics study of ciprofloxacin in malnourished children.

## 1 Introduction

Ciprofloxacin is a fluoroquinolone antibiotic that is being increasingly used in children with suspected Gram-negative infection. It exhibits a broad-spectrum activity against both Gram-positive and Gram-negative bacteria [1]. In general, fluoroquinolones exhibit concentration-dependent bacteriocidal activity, which depends on the ratio of maximum drug concentration (*C*_max_) to minimum inhibitory concentration (MIC) [2]. The ratio of the area under the concentration–time curve from zero to 24 h (AUC_0–24 h_) of fluoroquinolones to MIC is the most important predictor of clinical cure [3]. Satisfactory AUC can be achieved by oral administration as the drug is rapidly absorbed in children without cystic fibrosis [4]. Oral absorption of ciprofloxacin, however, can be significantly reduced by concomitant administration of milk and foods containing divalent and trivalent cations, such as Ca^2+^ and Al^3+^ [5–7]. Ciprofloxacin–food interactions may result in changes both in the rate and extent of absorption [8] and potentially lead to sub-therapeutic concentrations of ciprofloxacin and therapeutic failure.

Little is known about the pharmacokinetics of oral ciprofloxacin in infants and children and there are no data on the pharmacokinetics of ciprofloxacin in children with severe malnutrition. The outcome of African children hospitalised with severe and complicated malnutrition remains poor with many centres reporting high case fatality rates [9,10]. Invasive bacterial infection complicates up to 25% of fatalities with Gram-negative septicaemia, constituting between 48 and 55% of invasive bacterial pathogens [10]. For the treatment of bacterial infection complicating severe malnutrition the use of oral formulations of effective antimicrobial treatments are attractive since they are both practical and affordable. It has been demonstrated in critically ill patients that the pharmacokinetics of ciprofloxacin exhibit large inter-individual variability [11,12]. Children with severe malnutrition present to hospital with a range of complications and co-morbid infections including HIV/AIDS, invasive bacteria infection and malaria which render them severely ill [13]. Clinical studies investigating the pharmacokinetics of ciprofloxacin children with severe and complicated malnutrition are urgently needed since the pharmacokinetic processes (absorption, distribution, metabolism and excretion) may be altered due to associated pathophysiological changes, necessitating rational dosage adjustment of antibiotics. Determination of plasma ciprofloxacin concentrations *in vivo* is a valuable pharmacological tool in optimizing drug dosage regimens. Therefore, there is a need to develop and validate a suitable bioanalytical method for measurement of ciprofloxacin concentrations in malnourished children to support clinical pharmacokinetic studies.

Various bioanalytical methods used to measure ciprofloxacin concentrations in biological matrices have been reported. They include capillary electrophoresis [14], spectrophotometry [15,16] and high-performance liquid chromatography (HPLC) coupled with ultraviolet (UV) [17–25], fluorescence [26–35], and both UV and fluorescence [36,27] detection. Recently, HPLC methods coupled with mass spectrometry (LCMS) for determination of ciprofloxacin in human plasma [27,38] have also been published.

However, most of these techniques have various limitations. Although LCMS offers excellent selectivity and sensitivity and faster analysis time than HPLC methods, it requires relatively expensive instrumentation and highly skilled technical expertise. These may not be readily available and/or affordable for most laboratories in resource-limited settings. In such settings, selective and sensitive HPLC methods are preferable to more expensive LCMS techniques. Moreover, some of the published methods involve lengthy, time-consuming and laborious sample pretreatment and clean-up procedures, including the use of expensive solid phase extraction (SPE) cartridges [16,34] and long chromatographic run times (≥10 min) [17,19,21,31,32,34,37,39,40]. In addition, some of the reported methods involve the use of large volumes (≥1000 μL) of plasma/serum samples [17,18,32,35,39] rendering them unsuitable for repeat sampling in children where blood sample volume must be kept to a minimum. Sample clean-up procedures often involve multiple extraction steps. Thus, the use of an internal standard (IS) is crucial to account for any loss of analyte. However, some of the reported methods do not include the use of an IS in the sample clean-up procedures, or involve addition of IS after sample extraction [20]. This may lead to variability in extraction recovery and consequently, inconsistent pharmacokinetic data. Ciprofloxacin displays strong fluorescence properties and thus more readily detected using a fluorescence detector without the need for lengthy derivatization procedures.

The aim of this study was to develop and validate a simple, selective and sensitive reverse-phase HPLC method coupled with fluorescence detection for the determination of ciprofloxacin in small volumes (200 μL) of human plasma. The validated method was successfully applied to a population pharmacokinetics study of oral ciprofloxacin (10 mg/kg) in children admitted to hospital with severe malnutrition.

## 2 Experimental

### 2.1 Chemicals and reagents

Ciprofloxacin (*Ciprobay*^*®*^; batch no. 1396107; purity ≥ 98%) was purchased from Sigma–Aldrich Chemie GmbH (Steinheim, Germany). Sarafloxacin hydrochloride trihydrate (*VETRANA*L^®^; batch no. 7204X; purity 97.2%; used as the IS) was bought from Riedel-de Häen (Sigma–Aldrich Laborochemikalien GmbH; Steinheim, Germany). Acetonitrile (HPLC-grade) was purchased from Fisher Scientific (Loughborough, Leiceistershire, UK). Methanol (*CHROMASOLV*^®^ grade) was obtained from Sigma–Aldrich Chemie GmbH (Steinheim, Germany). Orthophosphoric acid (85%) and triethylamine (both HPLC grade) were bought from BDH Laboratory Supplies (Poole, UK). Deionised water was prepared in-house using a commercially available water purification system (*Pur1te Select™* NCP; Pur1te Ltd., Oxford, UK). Drug-free human plasma was obtained from the National Blood Transfusion Service (Nairobi, Kenya).

### 2.2 Equipment

Chromatography was performed using a *SpectraSystem*™ HPLC system (Thermo Separation Products, San Jose, CA, USA), equipped with a binary gradient pump (P2000), a dual monochromator fluorescence detector (FL3000), and a vacuum degasser (SCM100). A Rheodyne^®^ manual syringe-loading valve injector (model 7125; Cotati, CA, USA) fitted with a 50 μL loop was used. Chromatographic peaks were recorded on a data integrator (*ChromJet* CH-1; Thermo Separation Products, San Jose, CA, USA). Column temperature was maintained at 40 °C using a column heater controller (model 7990; Jones Chromatography Ltd., Wales, UK).

### 2.3 Chromatography

Chromatographic separations were performed on a *Synergi*^®^ *Max-RP* analytical column (150 mm × 4.6 mm i.d., 4 μm particle size; Phenomenex Inc., Macclesfield, Cheshire, UK), protected by a guard column (*LiChroSpher*^*®*^ 100 RP-18e, 10 mm × 4.6 mm i.d., 5 μm particle size; Merck, Darmstadt, Germany), maintained at 40 °C, using a thermostatically controlled column heater. The mobile phase comprised a mixture of aqueous orthophosphoric acid (0.025 M)/methanol/acetonitrile (75/13/12%, v/v/v) and the pH was adjusted to 3.0 with triethylamine. The flow rate was set at 1.0 mL/min. The mobile phase was degassed by ultrasonication prior to use. The fluorescence detection wavelengths were set at 278 nm (excitation) and 450 nm (emission).

### 2.4 Preparation of standard solutions

A stock standard solution (1 mg/mL) of ciprofloxacin was prepared in 0.2 M hydrochloric acid (HCl). Working standard solutions (100 and 10 μg/mL) were prepared by serial dilution of the stock standard solution with 0.2 M HCl. A stock solution (1 mg/mL of the free base) of sarafloxacin (IS) was prepared by accurately weighing an appropriate amount of sarafloxacin hydrochloride trihydrate and dissolving it in methanol. Working standard solutions (100, 10 and 1 μg/mL) were prepared by making appropriate serial dilutions of the IS stock solution in methanol. The standard solutions were stored at −15 °C to −25 °C, protected from light and were used within one month.

### 2.5 Preparation of calibration standards and quality control samples

Quality control (QC) samples were independently prepared by spiking drug-free plasma with various amounts of ciprofloxacin to give ciprofloxacin concentrations corresponding to the low (LQC; 0.2 μg/mL), medium (MQC; 1.8 μg/mL) and high (HQC; 3.6 μg/mL) levels of the calibration curve. Aliquots (500 μL) of the QC samples were stored at −70 °C to −90 °C until analysis, and were thawed to room temperature before being analyzed together with the patient plasma samples.

### 2.6 Sample preparation

Ciprofloxacin and IS were extracted from plasma using protein precipitation based on a published method by De Smet et al. [37]. To each aliquot (200 μL) of plasma (calibration standards, QC samples or patient samples) in a 10 mL glass tube, was added the IS (200 ng; 20 μL of 10 ng/μL sarafloxacin) solution made up in methanol. Acetonitrile (2 mL) was then added to precipitate the plasma proteins. The samples were mixed by vortexing (2 min), and then centrifuged (4000 rpm for 10 min at 4 °C). The upper organic layer was transferred into a clean glass tube and evaporated to dryness in a water bath (40 °C) under a gentle stream of white spot nitrogen gas (BOC Kenya Limited, Nairobi, Kenya). The residue was reconstituted in mobile phase (100 μL), mixed by vortexing, and an aliquot (50 μL) injected onto the HPLC column.

### 2.7 Method validation

Method validation was carried out according to the USA Food and Drug Administration (FDA) bioanalytical method validation guidelines [41]. The HPLC fluorescence method was validated for selectivity, sensitivity, linearity, precision and accuracy, recovery, and stability.

#### 2.7.1 Selectivity

Selectivity of the assay method was assessed by evaluating potential interference from endogenous compounds and other drugs which are routinely co-administered with ciprofloxacin. Six randomly selected blank plasma samples were analyzed under the optimized chromatographic conditions described. The separation of ciprofloxacin and IS and probable endogenous compounds from plasma were checked by comparing the chromatograms of QC samples with that of drug-free blank plasma, in order to evaluate interference at the retention times of ciprofloxacin or IS. In addition, interference of drugs that are frequently co-administered with ciprofloxacin was tested, including paracetamol and chloramphenicol. These drugs were spiked in blank plasma samples at therapeutic concentrations of each drug. The samples were extracted and analyzed as described in Section 2.6. The peak and the retention time for each drug under the chromatographic conditions of the ciprofloxacin assay were recorded to assess whether the peak for co-administered drug co-eluted with that of ciprofloxacin or the IS.

#### 2.7.2 Limit of detection (LOD) and lower limit of quantification (LLOQ)

In order to estimate the LOD and LLOQ for the assay, drug-free plasma sample was extracted and six replicates were injected and analyzed according to the optimized chromatographic conditions. Further drug spiked plasma samples were injected. The LOD was defined as the lowest concentration of ciprofloxacin in spiked plasma, which produced a peak with as a signal-to-baseline noise (S/N) ratio of ≥3, using a using a 200 μL plasma sample. The LLOQ was defined as the lowest concentration on the calibration curve which produced a peak with a S/N ratio of ≥10.

#### 2.7.3 Linearity of calibration curves

Calibration curves for ciprofloxacin were constructed using nine calibrators over a concentration range of 0.02–4.0 μg/mL (i.e., 0.02, 0.05, 0.1, 0.2, 0.5, 1.0, 2.0, 3.0 and 4.0 μg/mL). To a plasma sample aliquot (200 μL) of each calibration standard in a 10 mL glass tube was added IS (200 ng). The samples were assayed using the method described in Section 2.6. Thirteen calibration curves constructed on separate days were analyzed to evaluate the linearity of the calibration curves. Peak area ratios (ciprofloxacin/IS) were plotted against the corresponding ciprofloxacin nominal concentrations. Least-squares linear regression analysis of the calibration data was performed using the linear equation *Y* = *mX* + *c*; where *Y* is the ciprofloxacin/IS peak area ratio, *X* is the ciprofloxacin concentration, and *m* and *c* are the slope and intercept of the calibration curve. The linearity of each calibration curve was evaluated from the slope, intercept and correlation coefficient (*r*^2^) of each curve. Unknown ciprofloxacin concentrations were calculated by interpolation using the linear regression equation.

#### 2.7.4 Precision and accuracy

The precision and accuracy of the method were evaluated by analysing the QC samples at three different concentrations described in Section 2.5. The intra-assay (within-day) precision and accuracy of the method were evaluated by analysing plasma aliquots of each QC sample (*n* = 6 for each level) on the same day. The inter-assay (between-day) precision and accuracy were determined by analysing each QC sample (in duplicate) on six different days. Intra-assay and inter-assay precision were expressed as the percentage RSD of the measured concentrations of the QC samples. Accuracy was expressed as the percentage relative error (R.E.%) for each QC level by comparing the nominal concentration and the estimated concentration determined after extraction. For acceptable intra- and inter-assay values, the precision should not exceed 15% except at the LLOQ, where it should not exceed ±15%. The accuracy should be within ±15% of the nominal concentrations, except at the LLOQ, where R.E.% should be ≤20% [41]. The correlation coefficient of the calibration curve was set at 0.99 or better.

#### 2.7.5 Extraction recovery

The extraction recovery of ciprofloxacin from plasma was determined by assaying aliquots (200 μL) of drug-free plasma samples spiked with low and high ciprofloxacin concentrations (1.0 and 3.0 μg/mL) and IS (200 ng). In another set of test tubes, equivalent ciprofloxacin concentrations were spiked into the extraction solvent. The samples were extracted according to the procedure described in Section 2.6. The extraction recovery was determined by comparing the peak area ratios (ciprofloxacin/IS) from a given concentration of ciprofloxacin spiked in blank plasma with the peak area ratios obtained for the same concentration of ciprofloxacin spiked in the extraction solvent. Percentage recovery = (peak area of ciprofloxacin concentration extracted from spiked plasma/peak area of ciprofloxacin concentration directly added to extraction solvent) × 100]. The recovery of the IS was evaluated at the concentration used in sample analysis (200 ng).

#### 2.7.6 Stability

Stability experiments should reflect the conditions likely to be encountered during sample transfer, handling and analysis. In our setting, clinical samples from patients are stored at −20 °C immediately after collection, before being transferred (on dry ice) to a −80 °C freezer for longer term storage. The samples are thawed and analyzed immediately. The stability of ciprofloxacin in human plasma was evaluated using spiked plasma ciprofloxacin concentrations at low (1.0 μg/mL) and high (3.5 μg/mL), which were prepared from a fresh stock solution. An aliquot of each sample was analyzed immediately. The other aliquots were stored at various temperatures (2–8 °C, −15 °C to −25 °C and −70 °C to −90 °C) for 7 and 30 days. Ciprofloxacin concentrations were determined on day 7 and day 30. The results were compared with those obtained on the first day of the stability testing. The percentage concentration deviation was calculated. For acceptance criterion of stability, the deviation of the measured concentrations of the stored samples compared to the freshly prepared samples should be within ±15%.

### 2.8 Application of the method to population pharmacokinetic study in malnourished children

The validated HPLC method was applied to evaluate the population pharmacokinetics of ciprofloxacin in 52 Kenyan children with severe malnutrition. The Kenya Medical Research Institute (KEMRI)/National Ethical Review Committee approved the study which was conducted in accordance ICH/Good Clinical Practice (GCP) guidelines [42]. Children were recruited after obtaining informed consent from the parents/guardians of the children. All children received the standard medical treatment for severe malnutrition. Children who were recruited into the study were given oral ciprofloxacin (10 mg/kg every 12 h for 24 h). Four venous blood samples (0.75 mL) were collected into lithium heparinized tubes before drug administration (pre-dose), and at pre-specified times over an initial 24-h period following drug administration. The blood samples were immediately centrifuged (2500 rpm; 10 min) to separate plasma which was stored frozen at −80 °C until analyzed. In this manuscript, we report the plasma ciprofloxacin concentration–time profiles for three patients.

## 3 Results and discussion

### 3.1 Optimization of HPLC fluorescence chromatographic conditions

The chromatographic conditions were optimized through several iterations of the method to achieve good resolution, symmetric peak shapes for both ciprofloxacin and IS, and short run time (<10 min) while achieving selectivity and sensitivity. In preliminary experiments, several types of HPLC columns were evaluated, including Phenomenex *Synergi Polar RP* (150 mm × 4.6 mm; 4 μm), Luna C18(2) (150 mm × 4.6 mm, 5 μm), and Gemini C18 (150 mm × 4.6 mm, 5 μm). However, the retention times of ciprofloxacin and IS were longer and the peak shape was unsatisfactory. After careful comparison, a Phenomenex *Synergi Max RP* (150 mm × 4.6 mm; 4 μm) column was finally used with a flow rate of 1 mL/min, which produced good peak shapes and allowed a chromatographic run time of les than 10 min.

Several factors affect the chromatographic analysis of ciprofloxacin in biological matrices. The composition of the stock solution is critical, since dissolving ciprofloxacin in acidified methanol results in esterification of the carboxylic acid group [43]. In the present study, we used 0.2 M HCl to prepare a standard stock solution of ciprofloxacin, since the drug is poorly soluble in water. The pH of the mobile phase is very critical because the p*K* value of ciprofloxacin is affected by the composition of the mobile phase. In the present study, the pH of the mobile phase was adjusted to 3.0 with triethyamine. Like most fluoroquinolones, ciprofloxacin exhibits strong spontaneous fluorescence properties without derivatization, which allows adequate sensitivity [44]. The excitation spectrum of ciprofloxacin shows two maxima at 279 and 326 nm, while an emission spectrum shows an optimal response with emission at 442 nm [37]. In this study, the fluorescence detector was set at excitation and emission wavelengths of 278 at 450 nm, respectively.

### 3.2 Extraction procedure

Plasma is a complex biological matrix which can hamper detection of drugs and their metabolites. Sample clean-up procedure is therefore often needed to remove plasma proteins and potential interferences by other endogenous compounds prior to HPLC analysis. The sensitivity and selectivity of the method can be limited by the clean-up procedures. Ciprofloxacin is a polar, water-soluble and amphoteric compound, which makes it difficult to extract from plasma by liquid–liquid extraction (LLE) and solid-phase extraction (SPE) procedures. To avoid loss of analyte due to clean-up procedures and increase recovery of analyte, the number of cleanup steps in a sample preparation procedure should be kept to a minimum. Protein precipitation is commonly used for fast sample clean-up and disrupting protein–drug binding. Various organic solvents (e.g., acetonitrile and methanol) and acids (e.g. perchloric acid, phosphoric acid, trifluoroacetic acid) were used as protein precipitants in previously published methods [17,21,22,31,32,37]. Protein precipitation with acetonitrile or methanol is a simple procedure. In the present method, acetonitrile was chosen as the protein precipitant because it resulted in good signal intensities and high extraction recoveries for both ciprofloxacin and IS, and relatively clean chromatograms under fluorescence detection. Compared to other sample preparation methods, the reported method is simple, thus reducing the time required sample clean-up. The IS corrected for variation in the extraction recovery of the analyte during the sample clean-up steps. Although evaporation and reconstitution steps are included, the sample clean-up method remains a simple method and allows high throughput analysis necessary for pharmacokinetic studies.

Another advantage is the speed of the assay which is more rapid than most other methods that require long run chromatographic times (>10 min). Finally, the sample preparation procedure is based on a simple protein precipitation step, thereby eliminating the need of performing multiple steps involved in SPE and LLE procedures for other methods. The HPLC separation procedure is simple and can be easily reproduced because it has been performed under isocratic conditions, using a simple mobile phase composition, comprising orthophosphoric acid/methanol/acetonitrile and triethylamine.

### 3.3 Chromatography

**Fig. 1:**
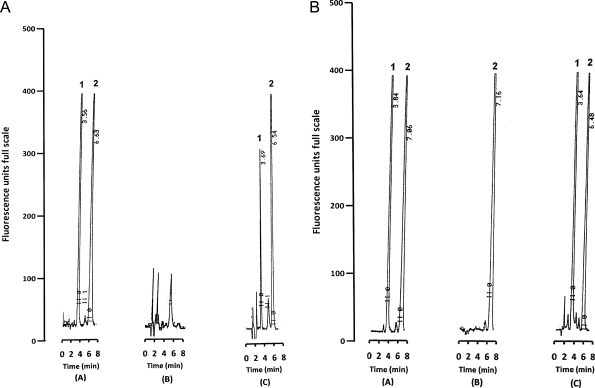
(a) Representative chromatograms of (A) un-extracted (direct injection) ciprofloxacin (100 ng) and sarafloxacin (internal standard, IS; 100 ng), (B) extracted aliquot (200 μL) of drug-free human plasma spiked with IS (200 ng) and (C) extracted aliquot (200 μL) of drug-free human plasma spiked with ciprofloxacin (0.05 μg/mL) and IS (200 ng). Peaks: 1 = ciprofloxacin; 2 = sarafloxacin (IS). (b) Representative chromatograms of extracted aliquot (200 μL) of (A) plasma sample spiked with ciprofloxacin (1.8 μg/mL) corresponding to the medium quality control (MQC) level and IS (200 ng), (B) a pre-dose plasma sample obtained following administration of an oral dose of ciprofloxacin (10 mg/kg every 12 h for 24 h) to a child with severe malnutrition and spiked IS (200 ng), (C) a plasma sample collected after 3 h following administration of oral ciprofloxacin (10 mg/kg every 12 h for 24 h) to a child with severe malnutrition, and spiked with IS (200 ng). The estimated plasma concentration of ciprofloxacin was 1.7 μg/mL. Peaks: 1 = ciprofloxacin; 2 = sarafloxacin (IS).

Under the described chromatographic conditions, ciprofloxacin was well separated from the IS, with retention times of about 3.6 min and 7.0 min, respectively (Fig. 1). The chromatographic run time for each sample was about 8 min. Fig. 1b showed chromatograms of plasma extracts from pre-dose (B) and 3 h (C) after administration of oral ciprofloxacin (10 mg/kg) to a child with severe malnutrition.

### 3.4 Method validation

#### 3.4.1 Assay selectivity

The selectivity of the method was tested by comparing the chromatograms of six different batches of blank human plasma. Fig. 1a shows a representative chromatogram of a blank plasma sample. There was no chromatographic interference from endogenous compounds at the retention times of ciprofloxacin and IS (Fig. 1a and b). The chromatographic peaks were well resolved to baseline. We investigated other co-administered drugs in spiked plasma which were not found to interfere with the assay.

#### 3.4.2 Linearity of calibration curves

The linearity of the calibration curve for ciprofloxacin in spiked drug-free plasma over the ciprofloxacin concentration range of 0.02−4.0 μg/mL was evaluated. Calibration curves consisting of nine concentration values (0.02, 0.05, 0.1, 0.2, 0.5, 1.0, 2.0, 3.0 and 4.0 μg/mL) of ciprofloxacin spiked in human plasma were constructed. The calibration curves for ciprofloxacin were linear over the range of 0.02–4.0 μg/mL. The mean linear regression equation of the calibration curves (*n* = 13) was: *Y* = 1.149*X* + 0.034; where *Y* represents the peak area ratio (ciprofloxacin/IS), and *X* represents the plasma concentration of ciprofloxacin (μg/mL). The mean correlation coefficient (*r*^2^) for the ciprofloxacin calibration curves was 0.998.

#### 3.4.3 Limit of detection (LOD) and lower limit of quantification (LLOQ)

The assay LOD was defined as the lowest concentration of analyte that produced a chromatographic peak distinguishable from the background noise (signal-to-noise (S/N) ratio 3:1). The LOD was 10 ng/mL, while LLOQ was 20 ng/mL. In some of the published HPLC methods, relatively large volumes of plasma (500–1000 μL) were used for sample preparation [17,18,32,35,39] so are less suitable for paediatric studies. In the present study only 200 μL of plasma was needed for the assay, so is suitable for population pharmacokinetic studies involving serial drug concentration measurements.

#### 3.4.4 Precision and accuracy

Intra-assay, inter-assay precision and accuracy data are summarized (Table 1). The intra-assay RSDs at 0.2, 1.8 and 16 μg/mL of ciprofloxacin were 7.7%, 4.3% and 2.8% (*n* = 6 in all cases), respectively. The inter-assay RSDs for the above concentrations were 5.7%, 3.6% and 4.9% (*n* = 6 in all cases), respectively. Intra-assay and inter-assay accuracy ranged from 93% to 105% (Table 1). These results indicated that the validated assay was precise, accurate and reproducible.

#### 3.4.5 Extraction recovery

The mean (±SD) percentage extraction recoveries of ciprofloxacin at the three ciprofloxacin concentrations (0.08, 1.8 and 3.6 μg/mL) were 78.1 ± 12.5% (*n* = 4), 83.5 ± 5.2% and 77.7 ± 2.0%, respectively (*n* = 8 in both cases). The mean (±SD) extraction recovery for IS was 94.5 ± 7.9% (*n* = 15) (Table 2). The use of an IS in the extraction procedure is crucial to compensate for variability in extraction efficiency. In the present study, sarafloxacin was chosen as the IS because ciprofloxacin and sarafloxacin are structural analogues, with similar chemical characteristics and properties. Sarafloxacin exhibits good fluorescence response at the excitation wavelength (278 nm) and emission wavelength (450 nm). It also displayed appropriate chromatographic retention with its peak sufficiently separated from that of ciprofloxacin, and relatively high extraction recovery. It is widely used in veterinary medicine, and therefore, the possibility of encountering interference from therapeutic concentrations in humans is minimal.

#### 3.4.6 Stability

Stability data for ciprofloxacin in spiked drug-free human plasma are summarized in Table 3. Ciprofloxacin was stable in spiked plasma when stored for one week at 2–8 °C, and for one month when stored at −15 °C to −20 °C and −80 °C to −90 °C.

### 3.5 Application of the method to population pharmacokinetic study in malnourished children

**Fig. 2:**
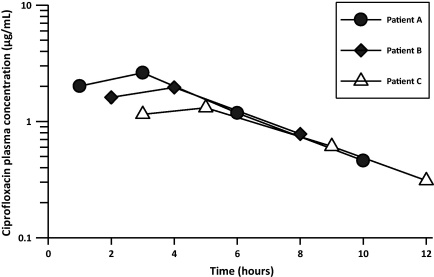
Semi-logarithmic plasma concentration–time profiles of ciprofloxacin following administration of an oral dose of ciprofloxacin (10 mg/kg every 12 h for 24 h) to three children with severe malnutrition.

To demonstrate its utility in a pharmacokinetic study, the method was applied to measure the concentrations of ciprofloxacin in plasma samples obtained from children with severe malnutrition who were given oral ciprofloxacin (10 mg/kg 12 hourly over 24 h). A representative chromatogram from an extracted plasma sample from a child is shown in Fig. 1b(B) and (C). There were no interfering endogenous peaks. The semi-logarithmic plasma ciprofloxacin concentration–time profiles of three patients are shown in Fig. 2. Oral absorption of ciprofloxacin was variable. Peak plasma ciprofloxacin concentrations ranging from 1.3 to 2.0 μg/mL were achieved within 1–5 h after oral administration of ciprofloxacin (10 mg/kg every 12 h) in children with severe malnutrition. In this group of children, ciprofloxacin was rapidly eliminated from the body, reaching undetectable levels within 12 h following drug administration (Fig. 2).

## 4 Conclusion

This paper describes a simple, rapid and selective, sensitive and reproducible method for the determination of ciprofloxacin concentrations in human plasma using HPLC coupled with fluorescence detection. The method offers several advantages for assaying the drug in low technology laboratories including ease, cost, good precision and accuracy with high sensitivity and selectivity. Moreover, the method requires only a small volume (200 μL) of plasma, which makes it suitable for studying the pharmacokinetics in young children. The sensitivity and simplicity of the method makes it suitable for routine therapeutic drug monitoring or clinical pharmacokinetic studies of ciprofloxacin. This assay method was successfully applied for a population pharmacokinetic study of ciprofloxacin following oral administration in children with severe malnutrition.

## Figures and Tables

**Table 1:** Intra-assay and inter-assay precision and accuracy of the assay for ciprofloxacin in plasma. SD = standard deviation; RSD = relative standard deviation; R.E. = residue error, calculated as [(estimated concentration − nominal concentration)/nominal concentration].

Quality control (QC) sample	Nominal concentration (μg/mL)	No. of replicates (*n*)	Mean (SD) estimated concentration (μg/mL)	Precision (%RSD)	Accuracy (%R.E.)
Intra-assay					
LQC	0.2	6	1.96 (0.02)	7.70	97.8
MQC	1.8	6	1.89 (0.08)	4.33	104.7
HQC	3.6	6	3.69 (0.11)	2.83	102.6

Inter-assay					
LQC	0.2	6	0.21 (0.01)	5.72	105.0
MQC	1.8	6	1.80 (0.07)	3.58	100.2
HQC	3.6	6	3.36 (0.16)	4.87	93.3

**Table 2:** Extraction recovery for ciprofloxacin and sarafloxacin (IS) from spiked plasma.

Analyte	Nominal concentration (μg/mL)	No. of replicates (*n*)	% Recovery (mean ± SD)	(%RSD)
Ciprofloxacin	0.08	5	72.8 ± 12.5	17.2
	1.8	8	83.5 ± 5.2	6.2
	3.6	8	77.7 ± 2.0	2.5
Sarafloxacin (IS)	200 ng	15	94.5 ± 7.9	8.3

**Table 3:** Stability of ciprofloxacin at various storage temperatures (2–8 °C, −15 °C to −25 °C and −70 °C to −90 °C).

Storage duration (days)	Ciprofloxacin concentration (μg/mL)	Storage stability (%)		
		2–8 °C	−15 °C to −25 °C	−70 °C to −90 °C
0	1.0	100.0	100.0	100.0
7	1.0	98.3	83.7	99.1
30	1.0	92.6	95.4	103.3
0	3.0	100.0	100.0	100.0
7	3.0	73.2	76.6	80.0
30	3.0	77.6	84.9	84.3
